# Toxicological and Functional Assessment of Minicell-Encapsulated dsRNA on Biocontrol Agents in Agriculture

**DOI:** 10.1021/acsenvironau.5c00067

**Published:** 2025-06-17

**Authors:** Mohammad Zarrabian, Lovely Adhikary, Mizuho Nita, Lahiri Sriyanka, Sherif M. Sherif

**Affiliations:** † Virginia Tech, School of Plant and Environmental Sciences, Alson H. Smith Jr. Agricultural Research and Extension Center, Winchester, Virginia 22602, United States; ‡ University of Florida, Gulf Coast Research and Education Center, Wimauma, Florida 33598, United States

**Keywords:** risk assessment, NTOs, IPM, BCAs, off-target

## Abstract

Double-stranded RNA (dsRNA)-based biopesticides represent promising tools for target-oriented pest and pathogen control. However, their compatibility with beneficial organisms used in biological control programs is not clear. In this study, the potential interactions between two dsRNA formulations—naked and minicell-encapsulated (ME-dsRNA)—and different commercialized bacterial, fungal, and insect biocontrol agents (BCAs) were examined. There was no toxicity of either of the two dsRNA formulations toward , , or , based on growth assays. ME-dsRNA significantly enhanced fungal BCA growth, likely due to improved uptake or protection. Coapplication trials on strawberry fruit and foliage showed that coapplication of dsRNA and BCAs was efficacious with no evidence of synergistic or antagonistic effects. Insect bioassays demonstrated that dsRNA sprays had no adverse effects on predatory mite populations ( and ). Additionally, in silico off-target analysis detected minimal potential matches in and , none of which corresponded to in vivo toxicity. Markedly, greenhouse and in vitro assays confirmed that neither dsRNA formulation interfered with the biocontrol efficacy of BCAs on in strawberry. Overall, this study provides strong evidence that dsRNA-based products, including ME-dsRNA, are compatible with key BCAs and pose minimal ecological risk.

## Graphical abstract

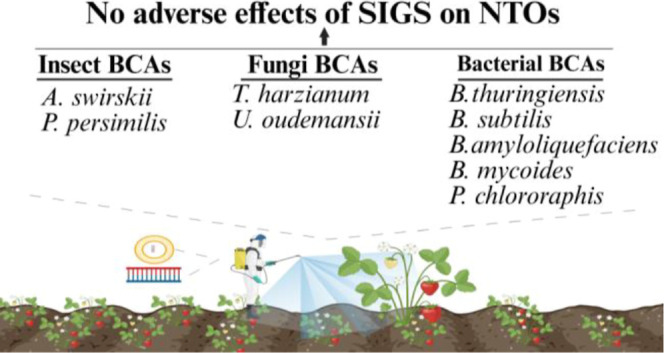

## 1 Introduction

The quest for innovative pesticides is a critical chapter in the ongoing story of sustainable pest management. This quest is becoming increasingly complex, with the escalating issue of developing pest resistance, risks to our environment and human health, increased pest pressure due to climate change, and increasingly stringent regulations.^1,2^ To address these concerns, RNA interference (RNAi) is a promising technology that has been gaining traction for decades and has received approval from the US Environmental Protection Agency (EPA).^3,4^ In RNAi-based pest and disease management, absorbed double-stranded RNA (dsRNA) triggers post-transcriptional gene silencing (PTGS) within the target pathogen or pest, effectively disrupting essential functions related to growth, development, and pathogenicity.^5^ This can be accomplished using two major strategies: host-induced gene silencing (HIGS) or spray-induced gene silencing (SIGS).^6^ Although both methods are highly efficient at controlling pests, they are accomplished using contrasting mechanisms. HIGS takes advantage of the plant’s own RNAi machinery,^7^ while SIGS uses the topical application of dsRNA or siRNAs to the plant surface.^8^ The potential of SIGS has been demonstrated in various systems, for example, application of a long noncoding dsRNA targeting fungal cytochrome P450 genes essential for ergosterol biosynthesis successfully protected barley from .^9^ In another instance, suppressing *AvCOPB2* and *AvProsbeta5* genes in through SIGS resulted in over 95% mite mortality with minimal leaf damage.^10^ It is this direct application that gives SIGS the significant advantage of being nontransgenic in nature, ruling out the genetic modification necessary in the case of HIGS.^11^

The effectiveness of SIGS hinges on the stability of the applied dsRNA, its efficient uptake by the target pest or pathogen, and the delivery of an appropriate dose.^12^ These factors make the selection of a suitable delivery method paramount for successful RNAi-based pest or pathogen control.^12^ To address these delivery challenges, several innovative strategies, including nanoparticle-based systems and nanovesicles, have been developed as dsRNA carriers.^13,14^ Among these, minicells—nondividing, anucleate vesicles derived from bacteria, typically 100 to 400 nm in diameter—show considerable promise. By encapsulating dsRNA, minicells provide a robust physical barrier that shields the nucleic acid from environmental nucleases, which would otherwise rapidly degrade unprotected molecules.^15,16^ This inherent protection enhances stability and persistence, particularly in challenging environments like plant surfaces, thereby increasing the likelihood of the dsRNA reaching its target organism intact.^17–19^ Moreover, this carrier, like others, offers a promising solution due to its capacity to carry substantial amounts of dsRNA, enabling targeted delivery in agricultural settings, regardless of dsRNA dosage, length, or molecular structure—advantages over naked dsRNA.^19–24^ However, minicell-mediated delivery strategy can also enhance environmental persistence of dsRNA,^19,25–27^ thereby potentially amplifying off-target impacts on nontarget organisms such as biocontrol agents (BCAs), beneficial microorganisms, soil microbiota, and also lead to the unintended presence or accumulation of these dsRNA molecules in water.^28,29^ Therefore, this study primarily aimed to investigate not only the effects of naked dsRNA but also, crucially, the impact of the novel minicell carrier—a delivery system with the potential to revolutionize agricultural dsRNA application.

As emphasized by Raybould and Burns,^28^ effective risk assessment for dsRNA-based pesticides requires a thorough understanding of the inherent properties of both the dsRNA molecules and the specific characteristics of the minicell delivery system, moving beyond simple on- or off-target categorization. This nuanced approach allows for a more comprehensive evaluation of potential impacts on both target and nontarget organisms. These risk assessment principles are already established within Integrated Pest Management (IPM) frameworks and could directly apply to dsRNA.^30^ For example, azole fungicides inhibit ergosterol biosynthesis in fungi—a crucial property for their antifungal activity, but one that also raises concerns about off-target effects in other organisms with similar pathways.^31^ Similarly, dsRNA’s inherent property is triggering RNAi. While the on-target effect is specific gene silencing in the target pest, this same property could unintentionally silence genes in nontarget organisms with sufficient sequence similarity. This is particularly crucial when combining dsRNA with BCAs, especially fungal BCAs, as it necessitates assessing the inherent properties of both components due to the potential for unpredictable interactions. Such a combined assessment is essential because the mixture can present risks distinct from those of individual agents.^32,33^ Indeed, recent research highlights the potential of dsRNA-based biopesticides as compatible and synergistic components within IPM strategies. Caccia et al. (2020), for example, showed that dsRNA can enhance the efficacy of by suppressing insect immune responses. However, dsRNA risk assessment, especially when combined with other factors, becomes complex and requires case-by-case evaluation.^34^

This complexity is amplified when multiple factors, such as dsRNA and its carriers, are present concurrently. While numerous studies suggest minimal effects of naked dsRNA on nontarget organisms,^35^ a significant gap remains in assessing alternative exposure pathways and the environmental safety of novel carriers, e.g., minicells, which require further investigation. This is particularly critical given the EPA’s requirement that pesticides pose no risk to humans or the environment for approval.^36^ Therefore, the overall goal of the present study is to investigate the effects of minicell-encapsulated dsRNA (ME-dsRNA) on the survival, growth, and performance of nontarget organisms, including bacterial, fungal, and insect BCAs. Our study specifically aimed to demonstrate the compatibility of ME-dsRNA with these BCAs, exploring its potential as a novel approach within IPM programs. Additionally, we investigated the reliability of in silico off-target predictions, demonstrating their dependence on data set selection, and importantly, not every off-target effect predicted by in-silico analysis occurs in living organisms (in vivo).

## 2 Methods and Materials

### 2.1 Experimental Organisms and Rationale

To evaluate the efficacy of various biological control agents, a study was conducted utilizing nine commercial products containing strain T-22, , , strain D747, isolate J, strain U3, strain AFS009, , and . These agents were selected for their common commercial application in biological control (see Table S1 for the source of these materials). Two key insects and fungi pests, (chili thrips), (two-spotted spider mites), and (a causal agent of Botrytis gray mold), were chosen as target organisms and strawberries (cv. Earliglow or Florida Brilliance) as host plants for this study.

### 2.2 In Vitro dsRNA Synthesis, Cloning, and Minicell Encapsulation Production

The dsRNA used in this study was designed similar to Islam et al.^19^ to suppress the pathogenic fungus by targeting the *DCL1* and *DCL2* mRNA transcripts, which are implicated in both fungal pathogenicity and growth.^37^ Following the in silico dsRNA design, *SacI* and *Pst*I restriction sites were incorporated at each end of the sequence. The sequence was synthesized as a gBlock by Integrated DNA Technologies (IDT, Coralville, IA, USA). The gBlock was cloned into the L4440 plasmid and transformed to (OmniMAX). Plasmids containing the target constructs served as templates for PCR reactions. Each 20 μL PCR reaction contained 2.5 ng of plasmid template, 0.5 μL Platinum SuperFi II DNA Polymerase (Thermo Fisher Scientific), 0.5 μL each of forward and reverse primers with T7 overhangs (10 μM), 1 μL dNTPs (100 μM), and 10 μL of 5X reaction buffer. PCR cycling conditions were initial denaturation at 95 °C for 3 min, 35 cycles of denaturation at 95 °C for 30 s, annealing at 60 °C for 30 s, and extension at 72 °C for 30 s, followed by a final extension at 72 °C for 5 min and storage at 4 °C. Eight microliters of the PCR products were used as templates for MEGAscript T7 transcription reactions (Thermo Fisher Scientific). dsRNA was then produced and purified according to the manufacturer’s instructions. Minicell-encapsulated dsRNA targeting the same genes in was provided by AgroSpheres (Charlottesville, VA), as previously described by Islam et al.^19^

### 2.3 Bacterial Growth and Viability Assessment

Bacterial BCAs were initially diluted according to the manufacturer’s recommendations. To minimize colony overlap, this solution was further diluted 500 times, and 50 μL was spread onto LB agar plates. Plates were incubated at 37 °C for all BCAs except , which was incubated at 28 °C. After 24 h, single colonies were selected and subcultured three times on fresh LB agar plates to ensure purity. For viability assessment purposes, 100 mL of overnight bacterial cultures were used to inoculate 20 mL of fresh LB media. When the culture reached an OD600 nm of 0.4, 1 mL was transferred to 15 mL of fresh LB broth and incubated in a rotary incubator shaker at 250 rpm and an appropriate temperature. When the culture reached an OD600 nm of 0.4, 1 mL of this culture was transferred into 14 mL of fresh LB broth that had been presupplemented with either naked-dsRNA (1000 ng/mL) or ME-dsRNA (2000 ng/mL), representing worst-case scenario concentrations. These inoculated cultures were then incubated in a rotary incubator shaker at 250 rpm and at an appropriate temperature. To assess microbial cell viability, 100 μL aliquots were collected from each culture at designated time points (0, 1, 3, 5, 7, and 9 h post-inoculation) and distributed into triplicate wells of a 96-well microplate for MTT assay. The MTT (3-(4,5-dimethylthiazol-2-yl)-2,5-diphenyltetrazolium bromide) assay was conducted using the Thermo Fisher Scientific assay kit, following the manufacturer’s instructions. Briefly, 10 μL of 12 mM MTT solution was added to each well and mixed thoroughly, followed by incubation at 37 °C for one and a half hours. To solubilize the MTT formazan crystals, 50 μL of DMSO (PhytoTech Laboratories, United States) was added to each well, mixed thoroughly, and the plate was incubated again at 37 °C for 10 min. After incubation, the plate contents were remixed, and absorbance was measured at 540 nm using a Synergy H1 microplate reader (BioTek, Oakville, ON, Canada). For all bacterial assays, fresh LB broth served as the positive control for bacterial growth, while hygromycin B (100 μg/mL) was used as the negative control. Additionally, LB broth medium supplemented with either minicell dsRNA or naked dsRNA (at the concentrations used in the experiments) but without BCAs was included to assess any background effects of the dsRNA preparations themselves.

### 2.4 Fungal Culture Preparation and Growth Assays

To isolate pure cultures of fungal BCAs from a commercial product, the manufacturer’s recommended dilution was further diluted 1000 times. This increased dilution was employed to minimize the density of fungal spores and prevent colony overlap on subsequent plates. The diluted suspension (50 μL) was then spread onto PDA plates. After 24 h of incubation at 25 °C, single, well-isolated colonies were selected. Each selected colony was subcultured onto fresh PDA plates three consecutive times to ensure purity. Conidial suspensions of and were prepared following the method described by Lazarotto et al.^38^ Briefly, surfaces of 14 day-old cultures were flooded with 10 mL of sterile distilled water, and the surface was gently rubbed to suspend conidia. The suspension was filtered through two layers of autoclaved cheesecloth to remove mycelia. The conidial suspension was then adjusted to 5 × 10^6^ conidia/mL (in autoclaved, sterile water) with the aid of automated cell counters (Countess 3 Automated Cell Counter (Invitrogen)). To assess the potential toxicity of naked and ME-dsRNA to fungal BCAs, we conducted two experiments using potato dextrose (PD) as a growth substrate. The initial experiment utilized what was considered a worst-case scenario dosage: 1000 ng/mL of naked dsRNA and 2000 ng/mL of ME-dsRNA, each prepared in autoclaved sterile water. The second experiment employed doses two times greater than these worst-case scenario concentrations for both naked and ME-dsRNA. The effect of naked and ME-dsRNA on fungal BCAs was evaluated using an MTT assay at 0, 6, and 24 hpi, following a method adapted from Li et al.^39^ Briefly, conidia were suspended in 10 mL of PD (broth) medium at a concentration of 1 × 10^5^ conidia/mL, combined with the appropriate concentration of naked or ME-dsRNA, and incubated in the dark at 25 °C with shaking at 100 rpm. At each time point, 500 μL aliquots of the culture were removed, and 50 μL of MTT reagent was added. The mixture was incubated for 1 h in the dark at 25 °C with shaking at 100 rpm. Following centrifugation (4000 rpm, 10 min) and removal of the supernatant, the resulting pellet was resuspended in 300 μL of DMSO and shaken for an additional 15 min at 25 °C in the dark at 100 rpm. For technical replicates, three 100 μL aliquots of the resulting solution were transferred to separate wells of a 96-well microplate, and the OD at 520 nm was measured by using a Synergy H1 microplate reader. The experiment was conducted with three replicates, and hygromycin B (150 μg/mL) and PD served as positive and negative controls, respectively. After measuring fungal BCA viability, 1 mL aliquots of the 24 h-old fungal culture were transferred to individual wells of a 24-well microplate and incubated in the dark at 25 °C for an additional 24 h. Fungal growth was then assessed visually. To further validate these findings, we repeated the entire experiment, excluding the MTT assay, using mycelial plugs. These plugs were taken from the edges of 7 day-old cultures. For treatment, each mycelial plug was incubated for 48 h in 1 mL of PD broth medium. This medium was supplemented with either naked dsRNA or ME-dsRNA at the respective ‘worst-case scenario’ concentrations previously described. Incubation occurred in the dark at 25 °C with 75% humidity.

### 2.5 Investigating the Synergistic Effects of dsRNA Formulas and BCAs

To investigate the potential synergistic effects of dsRNA with various BCAs, we designed two separate experiments targeting both strawberry leaves and fruits. From the ten BCAs investigated, we selected three strains known for their efficacy against : strain T-22, strain U3, and strain D747.^40–42^ In the first experiment, strawberry plants were cultivated in a greenhouse under controlled conditions: 16 h days (22 ± 1 °C) and 8 h nights (18 ± 1 °C) with 75 ± 1% relative humidity. Plants were grown in 1-gallon pots filled with a potting mix amended with 10% topsoil. Each treatment involved a combination of BCAs and dsRNA, applied to the leaves at the following concentrations: 2.5 × 10^8^ spores/ml for fungal BCAs, 1 × 10^10^ CFU/mL for , and twice the recommended rate for naked- (1000 ng/mL) and ME-dsRNA (2000 ng/mL). In the second experiment, mature and healthy strawberry fruits were purchased from a local farmer’s market. Twelve unwounded fruits of similar size were surface-sterilized with 5% sodium hypochlorite for 5 min, followed by four rinses with distilled water. A mycelial plug (7 mm) of was then inoculated onto each fruit. For separate leaf assays, a mycelial plug was also applied to each leaf of strawberry plants grown in the greenhouse. In both experiments, control treatments included water- and copper-based fungicide CUPROFIX FLEX (UPL NA Inc., North Carolina, USA). To assess the impact of treatments on leaves and fruits, the extent of necrotic tissue was quantified using ImageJ software.^43^

### 2.6 Evaluation of RNAi-Based Fungicides on Predatory Mite Populations

To assess the impact of RNAi-based fungicides on the predatory mites and , a greenhouse study was conducted at the University of Florida, Gulf Coast Research and Education Center. Strawberry plants (cultivar Florida Brilliance) with 4–5 expanded trifoliate were infested with either chili thrips () or two-spotted spider mites () from laboratory colonies whose maintenance adhered to protocols outlined in Kaur et al.^44^ and Busuulwa and Lahiri.^45^ Pest populations were allowed to establish for 7 days before the introduction of predators. Four hours after predator introduction, biofungicides were applied at a worst-case scenario concentration (1000 ng/mL for naked dsRNA and 2000 ng/mL for ME-dsRNA) via a foliar spray until runoff. Control plants received comparable applications of sterile water. Plants infested with were treated with the predatory mite , while those infested with were treated with . A randomized complete block design with six replications was employed, and the populations of pests and predators were monitored at weekly intervals for 3 weeks post-treatment. The experiment was repeated twice to enhance the robustness of the findings.

### 2.7 Data Analysis

Statistical analysis, including ANOVA to determine significant treatment differences and subsequent pairwise comparisons using the least significant difference (LSD) test, was performed in R (version 4.2.2). To assess potential off-target effects, a 398 bp dsRNA sequence was in silico fragmented into all unique 21-bp sequences using R (version 4.2.2) (Table S2). These fragments were then subjected to a BLAST search against a known database of BACs obtained from the National Center for Biotechnology Information (NCBI). The BLAST search results were subsequently filtered and analyzed against specific criteria, as detailed in the Results Section 3.5, to identify and evaluate potential off target matches.

## 3 Results and Discussion

RNAi-based strategies offer a promising avenue for sustainable and targeted pest and pathogen control.^46^ However, while transgenic dsRNA expression faces limitations in both feasibility and public acceptance, topical application (e.g., SIGS) using conventional methods has gained considerable traction. A prime example is Ledprona, a recently approved dsRNA-based SIGS product. Produced using Greenlights Biosciences’ cell-free bioprocessing platform, Ledprona effectively targets the Colorado potato beetle and has demonstrated efficacy in laboratory, greenhouse, and field settings.^47,48^ Despite such advances in SIGS technology, a significant challenge remains: naked dsRNA’s inherent environmental instability, which limits its field effectiveness.^23,49^ This limitation therefore highlights the critical need for enhanced delivery approaches. In this context, nanocarriers, e.g., cationic polymers, liposomes, and peptides, emerge as a promising strategy. They can improve dsRNA delivery, bioactivity, and persistence, as evidenced by recent studies.^50–53^ This is further supported by prior work of Islam et al., 2021, on minicells as biogenic vesicles, highlighting the efficient protection and delivery of dsRNA by minicells as key to enhanced RNAi efficacy under greenhouse conditions compared to naked dsRNA formulas. The successful field application of such technologies will ultimately depend on both their efficacy and environmental safety, including dsRNA stability under field conditions. While our current study does not specifically address the broader environmental instability of dsRNA formulations in the field, it aims to contribute to the environmental safety assessment by investigating the effects of this novel minicell delivery system on ecologically important nontarget organisms. This provides crucial information for the future of RNA-based pests and pathogen control in agriculture.

### 3.1 Selection of dsRNA Exposure Concentrations

The dsRNA concentrations employed in this study were selected to represent a ‘worst-case scenario’ for potential nontarget organism exposure. This strategy involved utilizing test concentrations (1000 ng/mL for naked dsRNA and 2000 ng/mL for ME-dsRNA) that are 2-fold higher than levels previously identified as effective against target organisms.^19^ The application of such elevated exposure levels is a common practice in toxicological assessments designed to rigorously evaluate potential adverse impacts on nontarget species under high-exposure conditions.^54–56^

### 3.2 Assessing the Interaction of Naked and ME-dsRNA Fungicide with Bacterial BCAs

Microorganism growth typically follows a predictable pattern, characterized by four distinct phases: a lag phase, a log phase (or exponential phase), a stationary phase, and a death phase.^57^ In fluctuating environments, each phase plays a critical role in population persistence. The lag phase is of significant interest due to its dual impact on bacterial populations: while it can enhance subsequent growth, it also renders cells more susceptible to external stressors.^58–60^ Therefore, a longer lag phase indicates enhanced adaptation to new environments.^61^ To assess the potential impact of the dsRNA-based pesticide, an MTT assay was conducted to monitor the growth kinetics and viability of several bacterial BCAs and (as a growth rate control) over 9 h. Most bacteria tested exhibited a short lag phase of approximately 1 h (Figures S1 and S2). However, strain AFS009, , and strain d747* displayed a more gradual increase in growth, characterized by a less steep slope in the growth curve preceding the log phase (Figure S1). Apart from the growth pattern, during the log phase, it is clear that at least for a one-time point, the growth rate of the bacteria exposed to dsRNA exceeded the control group, as was the case for naked dsRNA treatment in or ME-dsRNA treatment in (Figure S1).

Moreover, a comparative analysis of the average bacterial growth rates at 9 hpi revealed that despite initial growth fluctuations, all bacteria species exhibited similar (e.g., strain d747*, , , and ) or significantly enhanced (e.g., isolate J and strain AFS009) growth compared to the control treatment when exposed to various formulations of the dsRNA (Figure 1). This observation is consistent with the hypothesis that microorganisms, particularly prokaryotes lacking eukaryotic RNAi pathways, may degrade dsRNAs and potentially utilize their breakdown products as sources of carbon and nitrogen.^5,62–64^ This ability to utilize nucleic acids as nutrients is further substantiated by recent research identifying a P-type ATPase transporter (*PtaT*) in as a potential mechanism for sequence-specific tsRNA uptake.^65^ Huang and colleagues showed that efficiently possesses the capacity to utilize DNA as both a carbon and nitrogen source, achieving this through the direct internalization and subsequent periplasmic digestion of long DNA molecules.^66^ Consistent with this capacity for nucleic acid utilization, we observed enhanced growth (specifically at 5 and 7 hpi in second trail (Figure S2)) in the presence of dsRNA. This growth enhancement was observed with both naked dsRNA and dsRNA delivered via minicells, indicating that the delivery method did not significantly impact the observed effect (Figure.S1). Overall, these findings suggest that the naked- or ME-dsRNA-based fungicide has no adverse effects on the viability of the bacterial BACs tested in this experiment.

**Figure 1:**
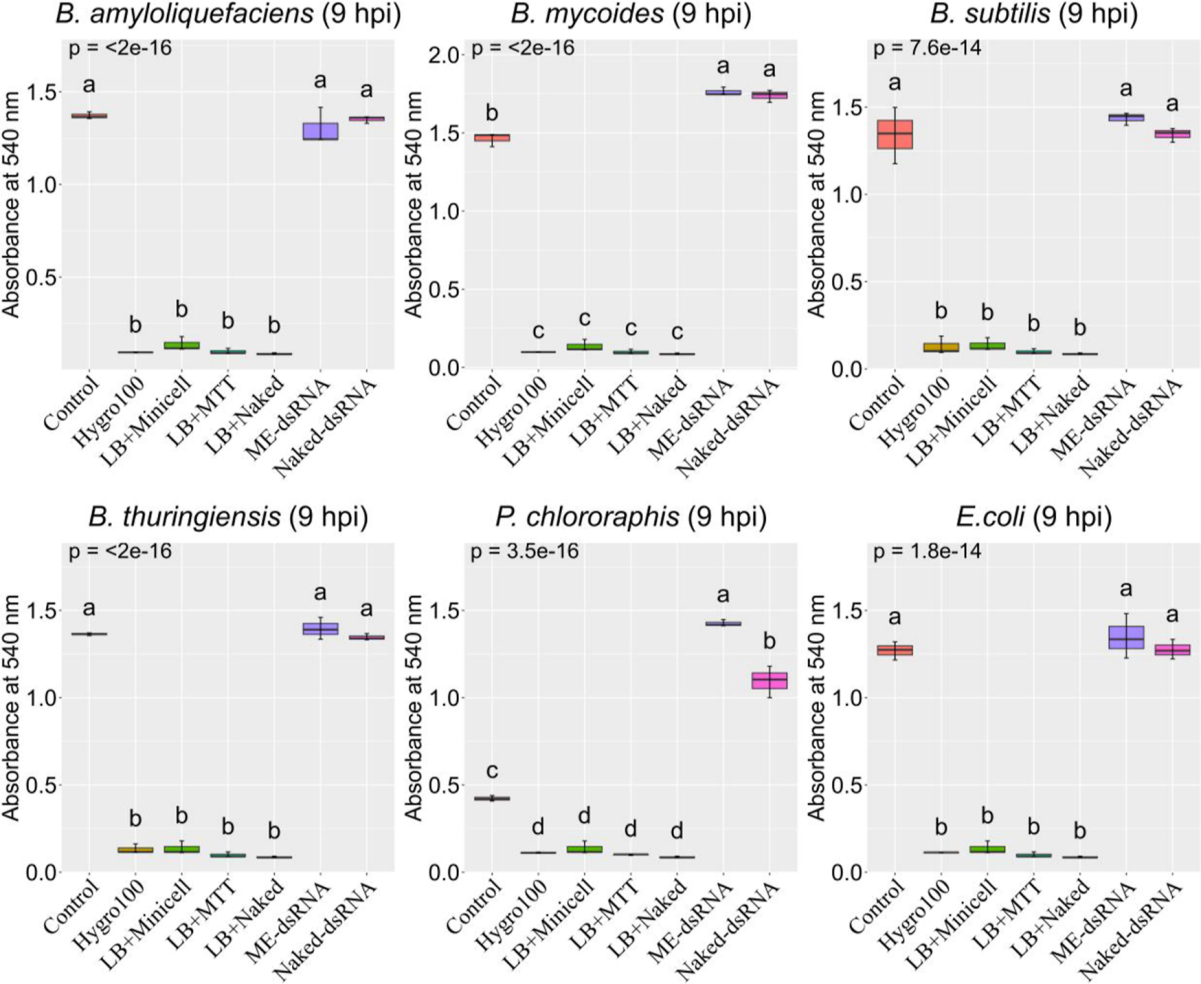
Viability of diverse bacterial biocontrol agents, inoculated by transferring 1 mL of culture (OD600 nm of 0.4) into 15 mL of fresh LB broth that was presupplemented with dsRNA formulations at worst-case scenario concentrations (1000 ng/mL for naked dsRNA and 2000 ng/mL for ME-dsRNA). Measurements were taken at 9 h post-inoculation. Viability was assessed by an MTT assay, measuring absorbance at 540 nm (*Y*-axis). Box plots display the distribution of absorbance values for each treatment group (*X*-axis). Groups within each panel sharing the same letter above the box plots are not statistically significantly different (*P* > 0.05, based on LSD following ANOVA). Each treatment group consisted of three biological replicates. Error bars represent the standard error of the mean (SEM).

### 3.3 Comparative Analysis of Fungal BCA Responses to Naked- and ME-dsRNA

To evaluate the impact of dsRNA-based fungicides on two fungal BCAs ( T-22 and U3), we conducted an MTT assay where fungal spores (10^5^ spores/mL) were exposed to either 1000 ng/μL naked dsRNA or 2000 ng/μL ME-dsRNA, and cell viability was assessed over 24 h (Figure 2). At each hpi, we found a statistically significant treatment effect (*P* ≤ 0.05). Initially (6 hpi), showed significantly higher viability with ME-dsRNA than with the control (PD), while naked dsRNA showed lower viability (Figure 2). By 24 hpi, ME-dsRNA treatments maintained their superior efficacy compared to the control. In contrast, naked dsRNA treatments, by this time point, showed viability levels comparable to those of the control (Figure 2).

**Figure 2:**
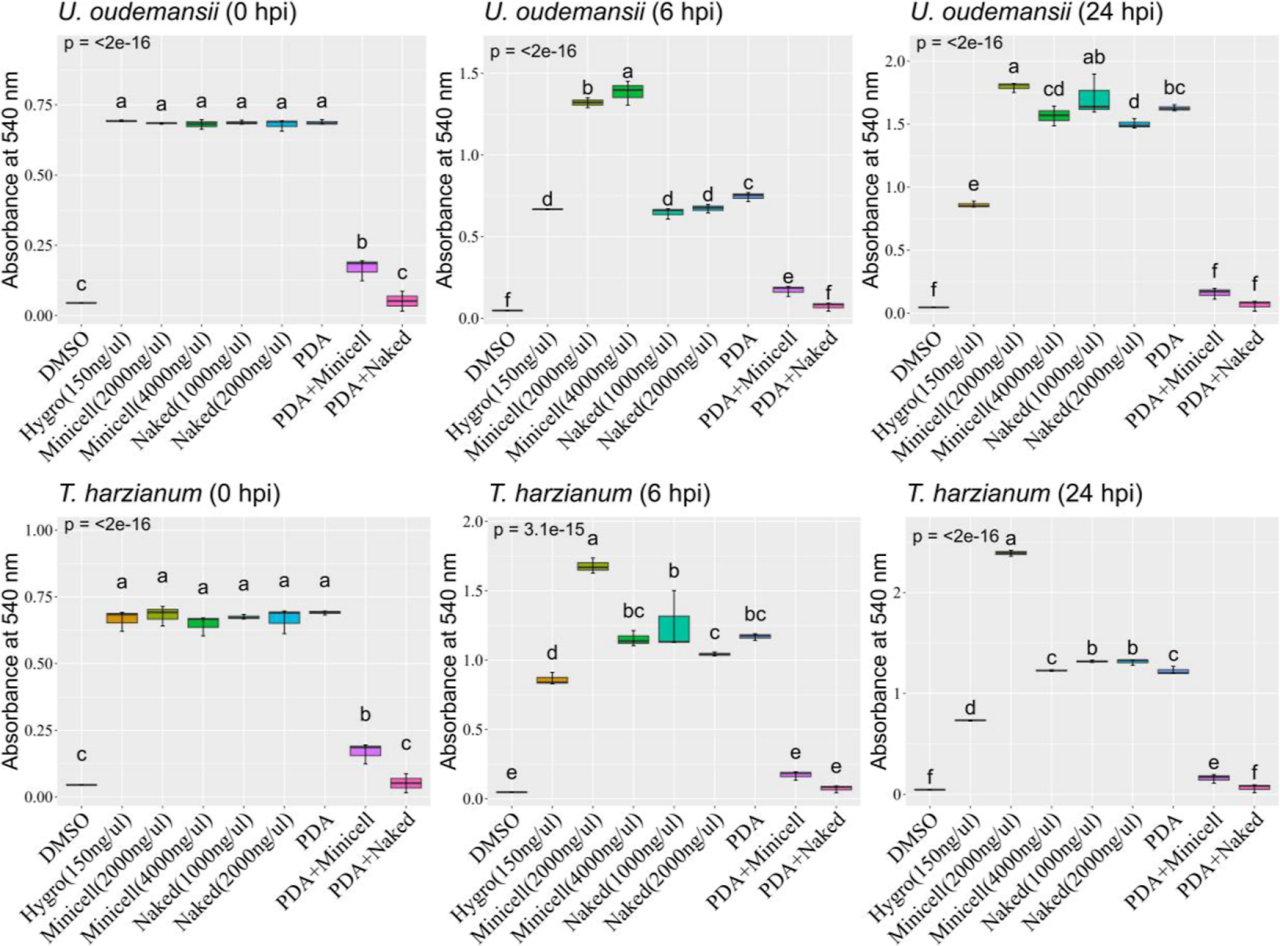
Growth dynamics of strain t-22 and strain u3 spores or mycelial plugs on the PDA medium exposed to naked or minicell-based dsRNA under the worst-case scenario (two or four times more than the recommendation). Box plots with identical letters denote statistically insignificant differences in treatment outcomes (*P* ≤ 0.05). Each treatment group consisted of three biological replicates. Error bars represent the standard error of the mean (SEM).

For , ME-dsRNA (but not naked dsRNA) significantly increased the viability over the control at 6 hpi. This advantage persisted at 24 hpi, when naked dsRNA also resulted in viability levels exceeding the control (Figure 2).

To explore whether dsRNA may induce growth retardation, we increased dsRNA concentrations to up to four times the recommended level. Under these conditions, again showed higher viability at 6 hpi with ME-dsRNA (4000 ng/μL) compared to the 2000 ng/μL treatment, although the overall trend remained similar (Figure 2). However, by 24 hpi, the effect of ME-dsRNA treatment on the viability was no longer significantly different from that of the control, while naked dsRNA treatment showed a significant reduction in viability (Figure 2).

To assess longer-term effects, 24 h fungal cultures were transferred to 24-well plates and incubated for an additional 24 h. This revealed no significant impact on fungal growth, even at elevated dsRNA concentrations (2000 ng/mL naked dsRNA, 4000 ng/mL ME-dsRNA), with only minor growth reduction observed for in elevated dsRNA concentrations (Figure 3).

**Figure 3:**
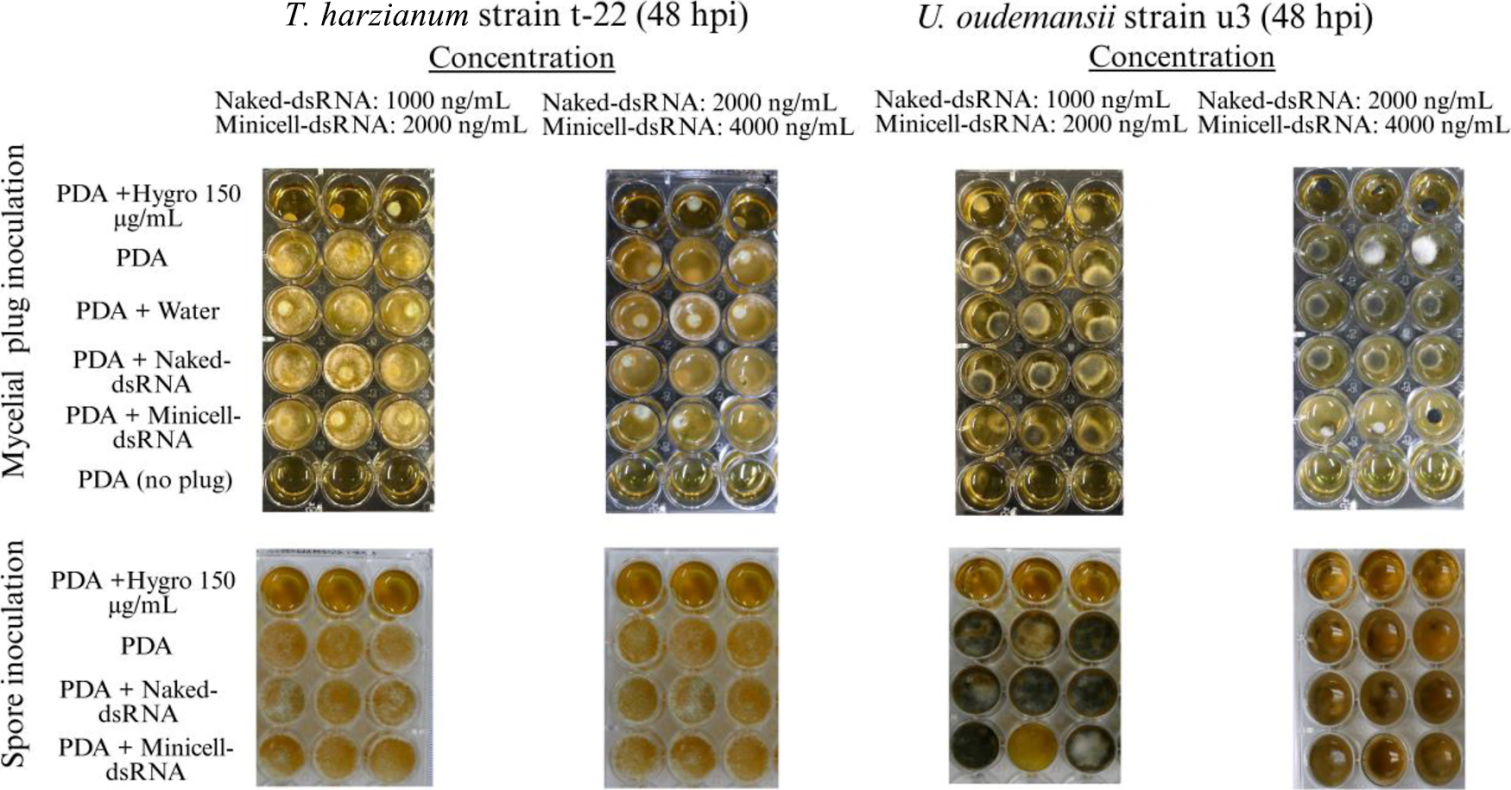
Growth dynamics of and spores or mycelial plugs on the PDA medium after 48 h of exposure to naked or ME-dsRNA under the worst-case scenario.

Finally, to compare the specificity of dsRNA and a common fungicide for , we evaluated the effects of copper-based fungicide (using a manufacturer-recommended concentration) on and . The results indicated that copper nonselectively inhibited the growth of all fungi tested (Figure S3). In contrast, neither naked nor ME-dsRNA affected the growth and survival of the two fungal BCAs examined (Figures 3and S3). This aligns with copper’s known broad-spectrum mechanism of action, which includes protein denaturation, reactive oxygen species generation, and membrane disruption.^67^

The enhanced growth observed in both fungal BCAs in the presence of dsRNA—particularly when encapsulated in minicells—supports the nutrient hypothesis, which proposes that dsRNA may act as a supplemental nutrient source for fungi.^66^ This working hypothesis is consistent with our results, where and exhibited significantly increased growth rates when treated with ME-dsRNA (Figure 2). However, the observed enhancement in fungal viability is likely multifactorial. Encapsulation may improve the bioavailability of dsRNA by protecting it from nucleolytic degradation and facilitating cellular uptake. While the precise mechanisms for minicell uptake by fungi are still being elucidated, several possibilities exist. Fungal cells are known to internalize particulate matter, and minicells, being bacterial-sized particles, could be taken up via endocytic pathways or phagocytosis-like mechanisms, which are documented in some fungal species.^68,69^ Additionally, the bacterial outer membrane components of the minicell might interact directly with the fungal cell wall or membrane, promoting adhesion and subsequent internalization or direct transfer of the dsRNA payload across the fungal cell envelope. These advantages are well documented for lipid-based nanoparticles—delivery systems that, like minicells, enhance solubility, permeability, and membrane interactions with fungal cells.^70–72^

By contrast, treatments with naked dsRNA led to a transient reduction in fungal growth, especially at 6 h post-inoculation (hpi). This could reflect lower initial uptake, particularly at early stages dominated by conidia, which are known to have thicker cell walls that impede macromolecular transport.^73^ As the fungi germinate and produce hyphae—with thinner and more permeable cell walls—naked dsRNA uptake likely increases.^74^ This shift may explain the increased growth observed at 24 hpi, which, in some cases, exceeded the control and aligns with the idea that dsRNA uptake and metabolism may support fungal proliferation under certain conditions.

The absence of enhanced growth at higher dsRNA concentrations, particularly with naked dsRNA at early time points, suggests that the relationship between dsRNA exposure and fungal growth is not solely nutrient-driven. Recent findings show that dsRNA can elicit canonical stress responses in fungi, in addition to activating RNA interference pathways.^73^ These sequence-independent stress responses, particularly prominent at higher concentrations, are transient and may represent an innate defense mechanism. Fungal cells may interpret dsRNA as a viral signal, triggering transcriptional reprogramming. For example, in Malassezia infected by a mycovirus, stress-related, translational, and phosphorylation-related genes are upregulated, while those involved in metabolism and cell division are suppressed.^75^ Similarly, fungal exposure to naked dsRNA could activate stress-related pathways, such as the HOG signaling cascade, thereby limiting early growth, while the organism reorients cellular functions to mitigate perceived environmental threats.

Minicell encapsulation may delay or prevent this stress recognition by shielding dsRNA from early detection. This could explain the more consistent growth observed in ME-dsRNA treatments across both BCAs, especially during the initial 24 h period. While high ME-dsRNA concentrations did not result in statistically significant reductions in viability compared to the control, growth at these concentrations was lower than that observed with lower ME-dsRNA doses (Figure 2), indicating a possible concentration-dependent threshold effect.

To determine whether RNAi-related off-target effects might also contribute to these observations, we conducted comprehensive BLAST analyses against the genomes of and (Section 3.5). In , we identified 49 potential mRNA cleavage targets. However, only one matched a gene encoding a carbohydrate esterase family 3 (CE3) protein, with the remainder corresponding to hypothetical proteins. Given that CE3 enzymes act on esterified carbohydrates such as xylan and pectin—which are not present in the potato dextrose (PD) medium used—this off-target interaction is unlikely to influence fungal growth.^76^ For , where a slight growth delay was observed with naked dsRNA, no significant BLAST hits were detected, further supporting the limited relevance of off-target effects under these experimental conditions.

Overall, the most parsimonious explanation for the observed growth patterns is that dsRNA acts primarily as a mild environmental stressor rather than as a toxicant. Fungi appear to temporarily divert metabolic resources to manage this stress, resulting in early growth delays that resolve once adaptation occurs, typically by 24 hpi. This convergence of growth rates across all treatments by 48 h (Figure 3) indicates that the stress response is short-lived and does not compromise long-term viability. The absence of toxicity, even at elevated dsRNA concentrations, and the ability of both fungal BCAs to maintain robust growth under these conditions suggest that neither dsRNA nor its minicell carrier is harmful to beneficial fungal organisms. These findings support the compatibility of dsRNA-based formulations with fungal BCAs and bolster their potential for integrated pest management strategies.

### 3.4 Comparative Analysis of Naked and ME-dsRNA in Combination with BCAs for 

BCAs offer a sustainable approach to managing plant pathogens, minimizing reliance on synthetic pesticides.^77^ Their adaptability, rapid growth, and pathogen specificity make them promising candidates for environmentally friendly pest control.^78^ However, integration with other control methods is often necessary for reliable, large-scale applications,^33^ requiring careful evaluation to avoid negative impacts on the BCAs themselves. Therefore, we selected three promising BCAs (, , and ), all of which have demonstrated efficacy against for further investigation in combination with various dsRNA fungicide formulations. To assess potential synergistic or antagonistic effects, we quantified necrotic lesions on strawberry leaves and fruits (Figures 4 and 5).

**Figure 4:**
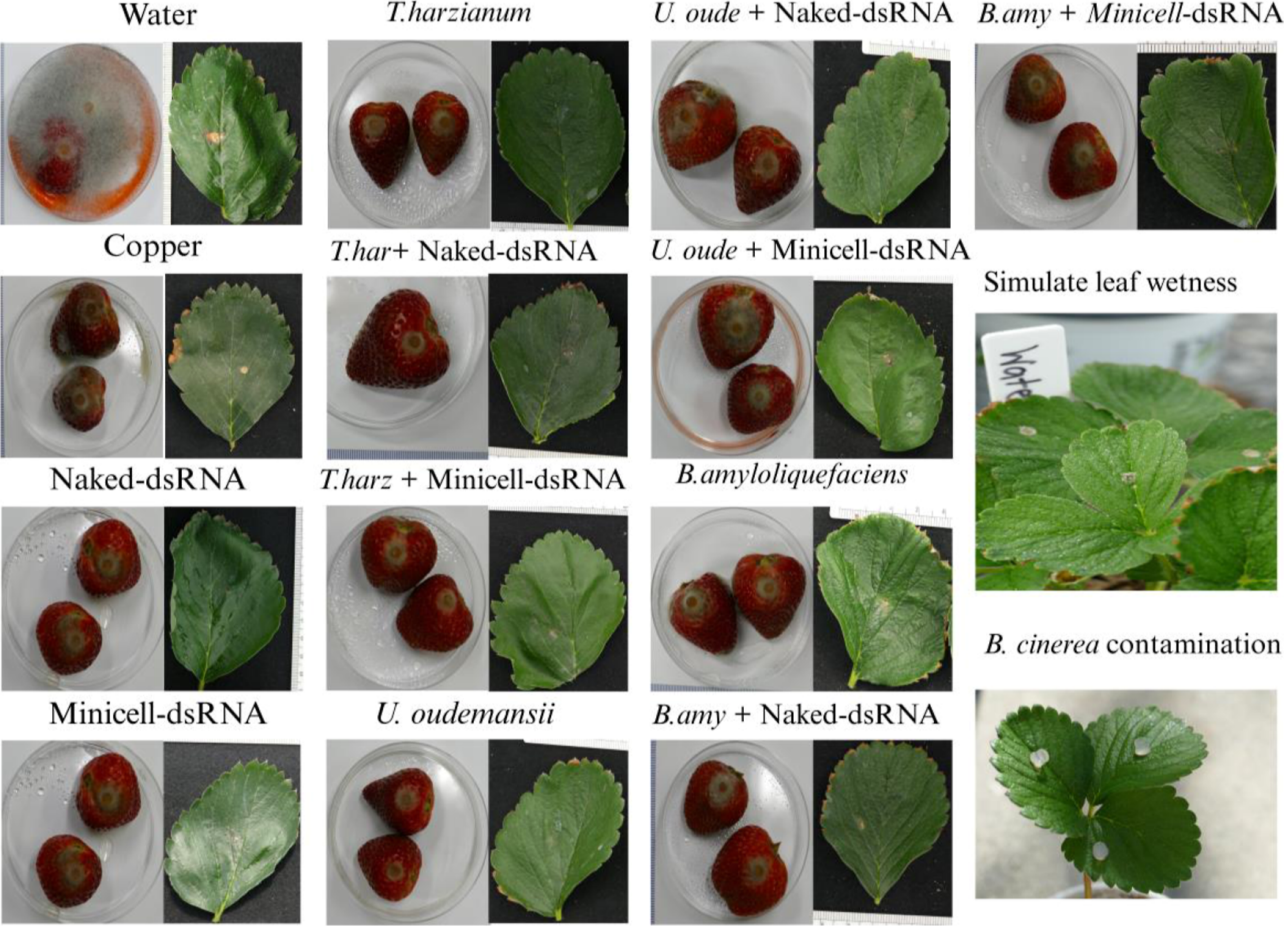
Effect of naked and ME-dsRNA and biocontrol agent treatments (, , and ), applied individually and in combination, on infection of strawberry leaves (21 dpi) and fruits (5 dpi). BCAs were applied at manufacturer-recommended concentrations, while dsRNA was used at the worst-case scenario (1000 ng/mL for naked dsRNA and 2000 ng/mL for ME-dsRNA).

**Figure 5:**
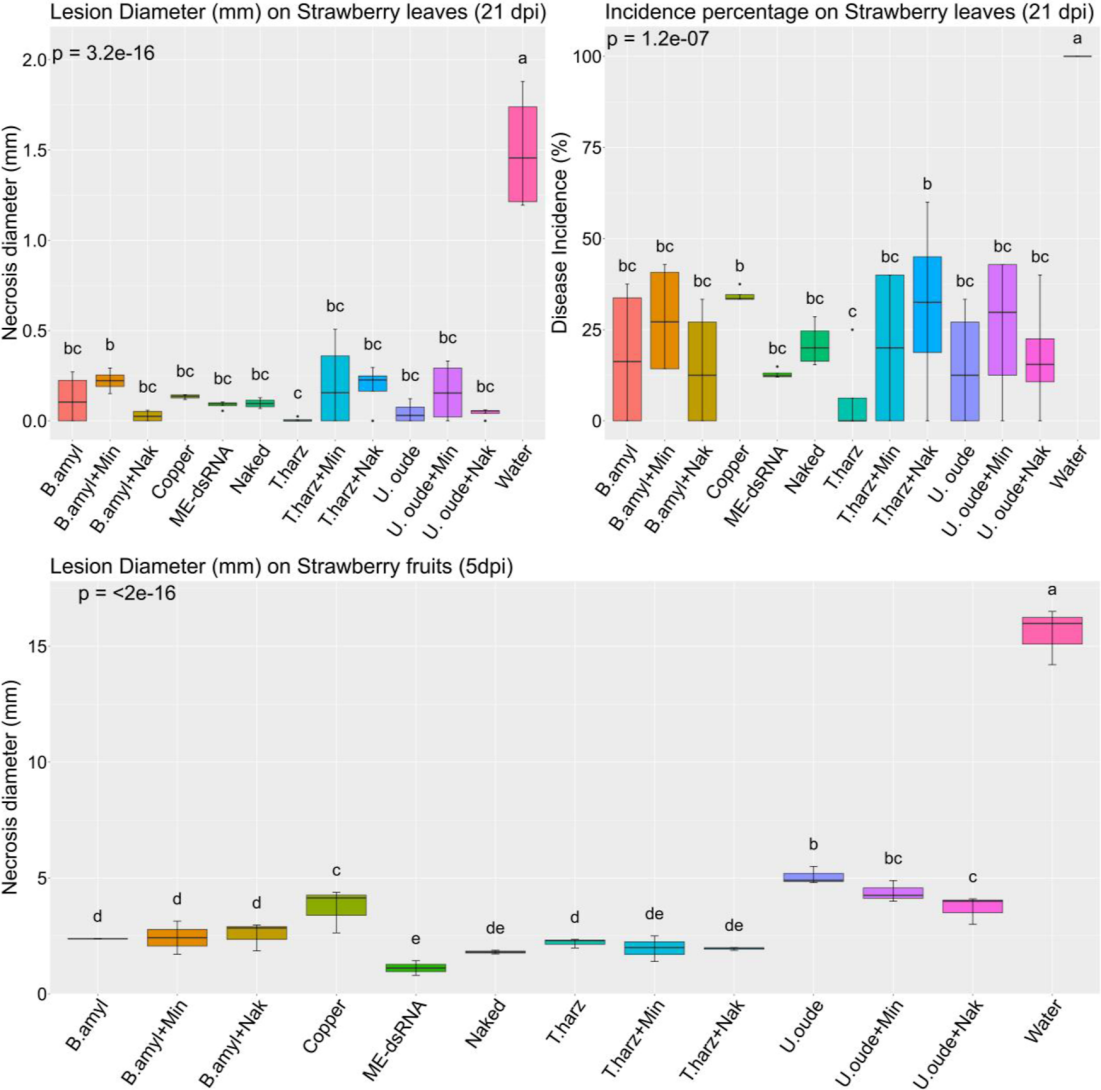
Comparative analysis of leaf necrosis and fruit infection diameter caused by on strawberries, followed by naked (1000 ng/mL) and ME-dsRNA (2000 ng/mL) treatment and biocontrol agents, both individually and in combination. Box plots with identical letters indicate statistically insignificant. Each treatment group consisted of three biological replicates, with three samples analyzed per replicate. Error bars represent the standard error of the mean (SEM).

The analysis indicated a significant divergence in all measured traits in leaves and fruits with the water control treatment for all other experimental groups. Beyond this, the only other significant differences observed were in the necrosis area between the + ME-dsRNA and treatments and in leaf incidence percentage between the + naked dsRNA and treatments (Figure 5). However, fruit samples exhibited a different pattern. Among the evaluated BCAs, , both alone and with dsRNA, and the copper-based fungicide showed the lowest efficacy against (Figure 5). This aligns with previous research indicating ’s limited effectiveness against Botrytis spp.^79,80^ Despite no significant difference between combined with naked dsRNA and ME-dsRNA in controlling , the combination of and naked dsRNA showed improved performance compared to alone. However, this improvement was inconsistent, appearing in only one trial. This finding is consistent with previous research demonstrating the synergy between and a chemical elicitor against Botrytis.^81^

Among the treatments tested, (alone and in combination with either naked or ME-dsRNA), naked dsRNA alone, and (alone and in combination with both dsRNA formulations) all demonstrated statistically similar efficacy and were significantly more effective than (alone or in combination) and the copper treatment. ME-dsRNA alone outperformed , , and copper, but its efficacy did not significantly differ from that of naked dsRNA or in either formulation. These findings are consistent with earlier reports regarding the individual efficacy of , , and dsRNA formulations.^19,42,80,82,83^

Despite the proven effectiveness of the individual treatments, no synergistic or antagonistic effects were observed when dsRNA was combined with BCAs on either leaves or fruits. This stands in contrast to the findings of Caccia et al.,^46^ who reported a synergistic interaction between dsRNA and . One possible explanation for the neutral effect in our study is the theoretical competition between BCAs and dsRNA for the interaction with the target site. However, this seems less likely given that the efficacy of the combined treatments did not significantly deviate from those of the individual agents. This pattern suggests that dsRNA and BCAs are likely to act through distinct and noncompeting mechanisms. While dsRNA primarily exerts its effects via RNA interference, it may also activate other plant defense pathways.^29^ In contrast, BCAs are known to suppress pathogens through multiple modes of action such as competition, mycoparasitism, antibiosis, and induced systemic resistance.^84–86^

An alternative explanation for the neutral interaction involves a subtle reduction in the BCA efficacy due to dsRNA exposure. Although dsRNA does not directly target the BCAs, it may exert mild, nonspecific inhibitory effects, such as triggering a stress response or minor off-target interactions. For instance, our earlier observation of a limited 21-bp sequence similarity (discussed in a later section) or previously discussed stress responses may contribute to this slight inhibition. Another possibility is that both BCAs and actively uptake dsRNA from the surrounding environment. Evidence from our cocultivation experiments, where the presence of dsRNA formulations enhanced growth in both bacterial and fungal populations (Figures 1, 2, and 3), supports this hypothesis. Therefore, another consequence of this uptake by BCAs could be a reduction in the dsRNA concentration available to target . Given that the antifungal efficacy of dsRNA is often dose-dependent,^19,87,88^ if BCAs actively sequester a portion of the applied dsRNA, the remaining concentration might fall below the threshold required for effective inhibition of . This uptake-based competition for the dsRNA resource, rather than a direct negative effect on BCA viability due to the dsRNA itself, could therefore diminish the overall antifungal outcome observed against and might partially explain the absence of a synergistic effect when BCAs and dsRNA were combined.

In summary, while the combined application of dsRNA and BCAs did not produce additive effects on disease control, it also did not result in any negative interactions. Given their distinct modes of action and demonstrated individual efficacy, the concurrent use of dsRNA and BCAs remains a viable strategy for integrated disease management.

### 3.5 Evaluating the Off-Target Effects and Efficacy of dsRNA in a Mite Predator–Prey System

This section examines the effects of different dsRNA delivery methods on the population dynamics of predatory mites ( and ) and their prey ( and ) across two independent trials. In both trials, a significant time effect was observed, with predator and prey populations declining from day 7 to day 21 (Table 1). This decline is consistent with established predator–prey dynamics, where prey depletion is expected to precede a subsequent decrease in predator populations.^89^

**Table 1:** Analysis of Variance (ANOVA) and Mean Comparisons of Pest and Predator Responses to Different dsRNA Formulations^a^ ^,^ ^b^

trail 1									
		S. dorsalis		A. swirskii		T. urticae		P. persimilis	
	Df	mean sq	*F* value	mean sq	*F* value	mean sq	*F* value	mean sq	*F* value
treatment	2	9.68	0.88^ns^	47.19	2.62^ns^	1044	5.5**	424.2	2.34^ns^
date	2	21.4	1.94^ns^	46.52	2.58^ns^	5398	28.47***	2385.8	13.41***
treatment/date	4	20.35	1.85	21.19	1.17	329	1.73	28.7	0.161
residuals	45	11		17.99		190		177.9	

comparison of mean values across time points				
	S. dorsalis	A. swirskii	T. urticae	P. persimilis
7 dpi	5.8	9.3	53^a^	37.4^a^
14 dpi	4	6.6	31^b^	41.1^a^
21 dpi	4	9.5	18.3^c^	19.6^b^

comparison of mean values across treatment				
	S. dorsalis	A. swirskii	T. urticae	P. persimilis
control	3.8	8	41.27^a^	37.7
naked-dsRNA	5.2	10.3	35.3^ab^	32.2
minicell-dsRNA	4.7	7.2	26.1^b^	28.11

trail 2									
		S. dorsalis		A. swirskii		T. urticae		P. persimilis	
	Df	mean sq	*F* value	mean sq	*F* value	mean sq	*F* value	mean sq	*F* value
treatment	2	128.39	6.38**	48.6	1.348^ns^	1376	5.4**	40.69	0.354^ns^
date	2	121.25	5.702**	1116.6	30.95***	5507	21.6***	280.02	2.43^**ns**^
treatment/date	4	104.33	4.9**	113.8	3.1*	549	2.1*	86.69	0.727^ns^
residuals	45	21.26		36.1		254		115.7	

comparison of mean values across time points				
	S. dorsalis	A. swirskii	T. urticae	P. persimilis
7 dpi	13.3^a^	21.7^a^	67.5^a^	26.8
14 dpi	9.2^b^	16.2^b^	41.2^b^	23.7
21 dpi	5.3^b^	6.1^c^	34.4^b^	19

comparison of mean values across treatment				
	S. dorsalis	A. swirskii	T. urticae	P. persimilis
control	10.5^ab^	15.4	55^a^	23.6
naked-dsRNA	7.6^b^	12.8	50^a^	21.5
minicell-dsRNA	12.94^a^	15.8	38^b^	24.38

a ns**:** not statistically significant. *: statistically significant at α = 0.05. **: statistically significant at α = 0.01. ***: statistically significant at α = 0.001.

b Mean comparisons without assigned letters indicate no significant differences among treatment levels, as determined by ANOVA analysis, and means with the same letters are statistically insignificant.

While statistically significant differences in populations were observed among treatments, particularly in the second trial (Table 1), these differences existed only between naked and ME-dsRNA. Crucially, neither naked nor ME-dsRNA treatment significantly affected populations compared to the untreated control (Table 1). For , the minicell-based dsRNA treatment significantly reduced populations, especially in the second trial, compared to naked dsRNA. This population reduction with the minicell formulation may be due to increased dsRNA protection from degradation (e.g., gut pH or enzymatic activity). This protection is particularly relevant given the off-target mRNA sequences we identified in during our in silico analysis (discussed below).

From the predator’s perspective, dsRNA treatments did not significantly influence predator populations across both trials (Table 1). This finding can be interpreted in two ways. First, direct exposure of predators to dsRNA during topical application had no significant effect, and second, despite observed prey population reductions (indicating dsRNA uptake), any transferred dsRNA, even if intact, which is unlikely given insects’ lack of dsRNA amplification machinery,^90,91^ did not significantly impact predator populations.

### 3.6 In Silico Analysis of Potential Off-Target Effects of Different dsRNA Formulations on NTOs

To investigate potential off-target effects of the dsRNA-based fungicide on six nontarget organisms (NTOs), including two pests, the 398 bp dsRNA fragment was in silico fragmented into all possible 21 bp sequences, yielding 378 unique fragments (Table S2). These fragments were then subjected to BLASTn analysis against the available NCBI nucleotide collection to identify potential sequence homologies. Results for organisms other than the bacterial BCAs are presented in Figure 5 and Tables S3–S9. Analysis of bacterial genomes was deemed unnecessary, as prokaryotes lack a canonical RNAi system,^92^ and, as shown previously, dsRNA is generally considered a nutrient source in these organisms. For our initial BLAST searches, we used species-specific Sequence Read Archive (SRA) databases whenever they were available. This was the case, for example, for the two insect predators that we examined. Strikingly, BLAST results varied considerably when using different databases, especially SRAs. For example, yielded 244,349 and 37,753 hits against SRR8266222 and SRR7851390, respectively (Table S10). This demonstrates the significant impact of database choice on off-target prediction, likely due to variations in experimental conditions, sequencing platform, library preparation, and sequencing depth.^93,94^ Consequently, all subsequent BLAST analyses used the NCBI core nucleotide database (core_nt).

Following in silico cleavage and BLAST analysis, a method for data analysis was developed. While complete in vivo generation of all possible 21 bp fragments is unlikely, the formation of a significant subset across different organisms remains plausible due to the variable cleavage specificities of Dicer enzymes.^95^ Although some Dicer enzymes exhibit preferential cleavage sites,^96,97^ their overall activity is largely stochastic. For instance, Asian corn borers produce numerous siRNAs with GGU nucleotide residues at the 5′- and 3′-ends, while red flour beetles exhibit more diverse cleavage sites (AAG, GUG, GUU).^97^ This lack of a universally defined nucleotide cleavage motif complicates in silico siRNA design. However, Chen et al.^98^ proposed classifying potential dsRNA off-target effects based on homologous sequence length and mismatches. They suggested that dsRNAs with greater than 80% sequence identity to target genes, which translates to at least a 16 bp perfect match or a 26 bp near-perfect match (with one or two mismatches), could trigger gene silencing. We adapted these criteria for siRNAs, requiring: (1) ∼80% overall similarity (∼16 nucleotides); (2) ≥18 bp similarity with one or two mismatches; (3) and for the final filtering step, we incorporated findings from Kobayashi et al.^99^ Their study indicated that the seed region (nucleotides 2–8) significantly influences both on- and off-target activities. Therefore, complete identity within this seed region was another criterion that we considered in our filtering process. These filtering results are presented in Figure 6 and Table S4.

**Figure 6:**
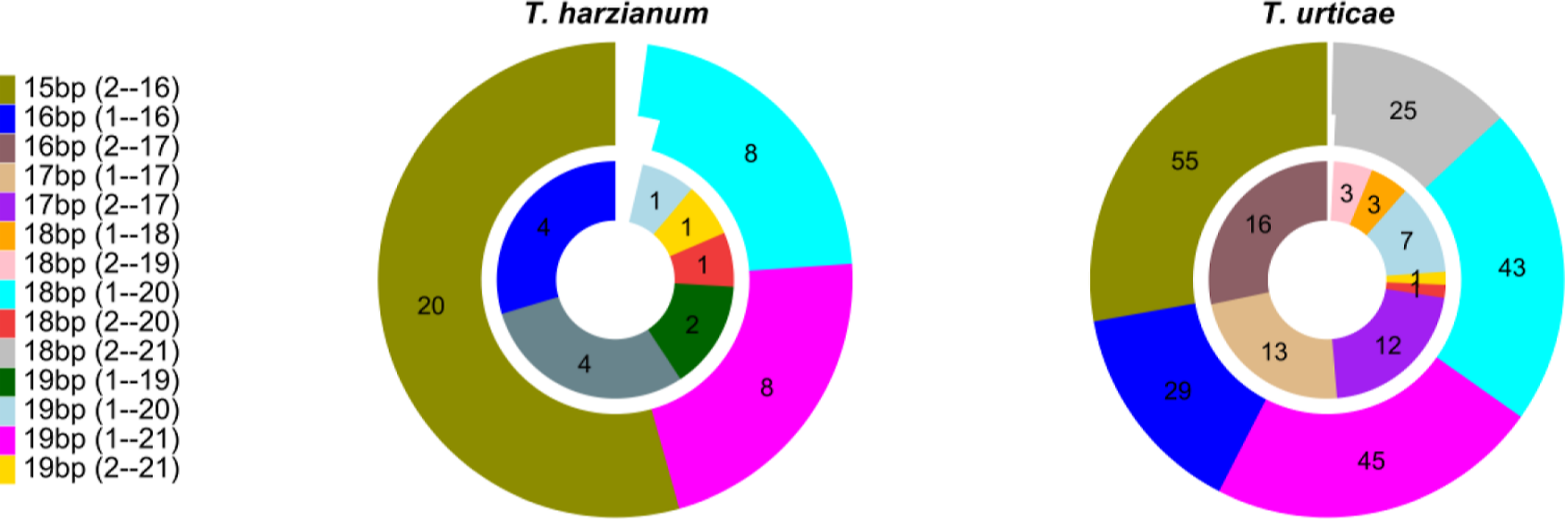
Pie chart illustrating the distribution of mismatches within 21-bp sequences derived from a 398-bp-long dsRNA. This analysis includes only those sequences meeting the following criteria: at least 15 bp of perfect match or at least 19 bp with one or two mismatches. In the figure description, number before ‘bp’ represents the number of perfectly matched nucleotides in the BLAST alignment. Number in parentheses indicates the start and end positions of the aligned region. Values in the pie chart show the number of BLAST hits meeting the criteria.

BLAST searches against the core nucleotide database (core_nt) revealed that most 21 bp fragments from , , , and showed limited sequence similarity (≤15 bp), and those with 16 or 17 bp similarity contained multiple mismatches (Tables S3, S5, S8–S10). Conversely, and exhibited numerous potential off-target binding sites, with 1.2% and 5.7% being ≥15 bp, respectively (Tables S3, S6, and S7). Among all these 21 bp sequences, after applying our criteria, only 49 and 253 potential off-target sites were identified for and , respectively (Table S3). For example, in , sequences 110 and 111 targeted mRNAs of microtubule-associated proteins, specifically regulator of microtubule dynamics protein 2. Silencing these mRNAs could be lethal because dynamic microtubules are essential for cell integrity and intracellular transport.^100^ Additionally, sequence 102 targeted mRNAs for nucleolar transcription factor 1 A and leucine-rich repeat flightless-interacting protein 2, which regulates toll-like receptor (TLR) signaling.^101^ These combined off-target interactions, particularly the disruption of microtubule dynamics, provide a plausible explanation for the in vivo toxicity of dsRNA observed in , as discussed previously. Similarly, also exhibited potential off-target binding sites. For example, sequence 125 showed homology to the mRNA encoding a carbohydrate esterase family 3 (CE3) protein. CE3 proteins are crucial for mycoparasitic fungi, facilitating the degradation of esterified carbohydrates in fungal cell walls, thus weakening the target pathogen and enhancing penetration.^76^ However, even if silencing of this nonspecific gene occurred in , it would likely not significantly impact its growth. On the other hand, this might explain the neutral effect observed when was combined with dsRNA to control .

Overall, our criteria appear to be positively useful for detecting potential off-target matches with mRNA and partially justify some of our results. However, it is important to consider that not all predicted siRNAs are likely to be produced in vivo. Furthermore, the Dicer cleavage of dsRNA is not entirely random. Research indicates that factors like structural position and RNA-binding specificity influence cleavage site selection by DCL enzymes.^61,102–104^ Consequently, a more in-depth investigation is essential to further develop and validate these criteria, especially concerning their application in vivo.

## 4 Conclusion

This study provides a comprehensive evaluation of the compatibility of naked and ME-dsRNA formulations with several commercially relevant bacterial, fungal, and insect BCAs. Across all tested BCAs, including , , and , neither formulation of dsRNA exhibited toxic effects at or above worst-case exposure levels. In many cases, particularly with ME-dsRNA, enhanced microbial growth was observed, consistent with the hypothesis that dsRNA may serve as a nutrient source or benefit from improved uptake via encapsulation. No long-term viability reduction or adverse physiological impacts were detected in fungal BCAs, and even at elevated concentrations, growth patterns generally recovered by 24–48 hpi. Additionally, coapplication of dsRNA and BCAs on strawberry leaves and fruits showed no evidence of antagonistic interaction. The absence of synergistic effects may be attributed to their distinct modes of action or potential competition for dsRNA uptake, as supported by observed growth increases in both BCAs and target pathogens during cocultivation.

The ecological safety of dsRNA-based fungicides was further supported by greenhouse trials with predatory mites ( and ), which revealed no significant impact on predator populations following the topical application of either formulation. Furthermore, in silico off-target analysis demonstrated that database selection significantly influences predicted dsRNA interactions, and while a few potential off-target matches were identified in and , these were not associated with notable in vivo toxicity in . Taken together, these results support the conclusion that both naked and minicell-encapsulated dsRNA are functionally compatible with a range of beneficial BCAs and pose minimal ecological risks under the tested conditions.

## Supplementary Material





## Notes

Growth kinetics of bacterial BCAs following exposure to naked and ME-dsRNA (Trials 1 and 2), assessment of copper fungicide specificity against two fungal BCAs, and comparative analysis of leaf necrosis and fruit infection diameter for the second trial (PDF)
Information on commercial biocontrol products used in this experiment, list of 21-bp sequences generated from 398-bp dsRNA used in this study, number of blast hits based on nucleotide matches against the query sequence, number of BLAST hits for different databases after applying criteria, and BLAST results of 21-bp fragments derived from 398-bp dsRNA against insect pests and fungal and insect BCAs (XLSX)
