# Supplementary material for: Toxicological and Functional Assessment of Minicell-Encapsulated dsRNA on Biocontrol Agents in Agriculture
Source: ACS Environ Au. 2025 Jun 17;5(4):427–41. doi: 10.1021/acsenvironau.5c00067 (PMC12272273; doi:10.1021/acsenvironau.5c00067)
Supplement: Supplementary file 1 [file vg5c00067_si_001.pdf]

# Toxicological and Functional Assessment of Minicell-Encapsulated dsRNA on Biocontrol Agents in Agriculture

Mohammad Zarrabian[1], Lovely Adhikary[2], Mizuho Nita[1], Lahiri Sriyanka[2], Sherif M. Sherif[1*]

1 Virginia Tech, School of Plant and Environmental Sciences, Alson H. Smith Jr. Agricultural Research and Extension Center, Winchester, VA, 22602, United States.

2 University of Florida, Gulf Coast Research and Education Center, Wimauma, FL, 33598, United States.

* Corresponding author: Sherif M. Sherif; E-mail address: ssherif@vt.edu; Tel: +1 (540)- 232-6035

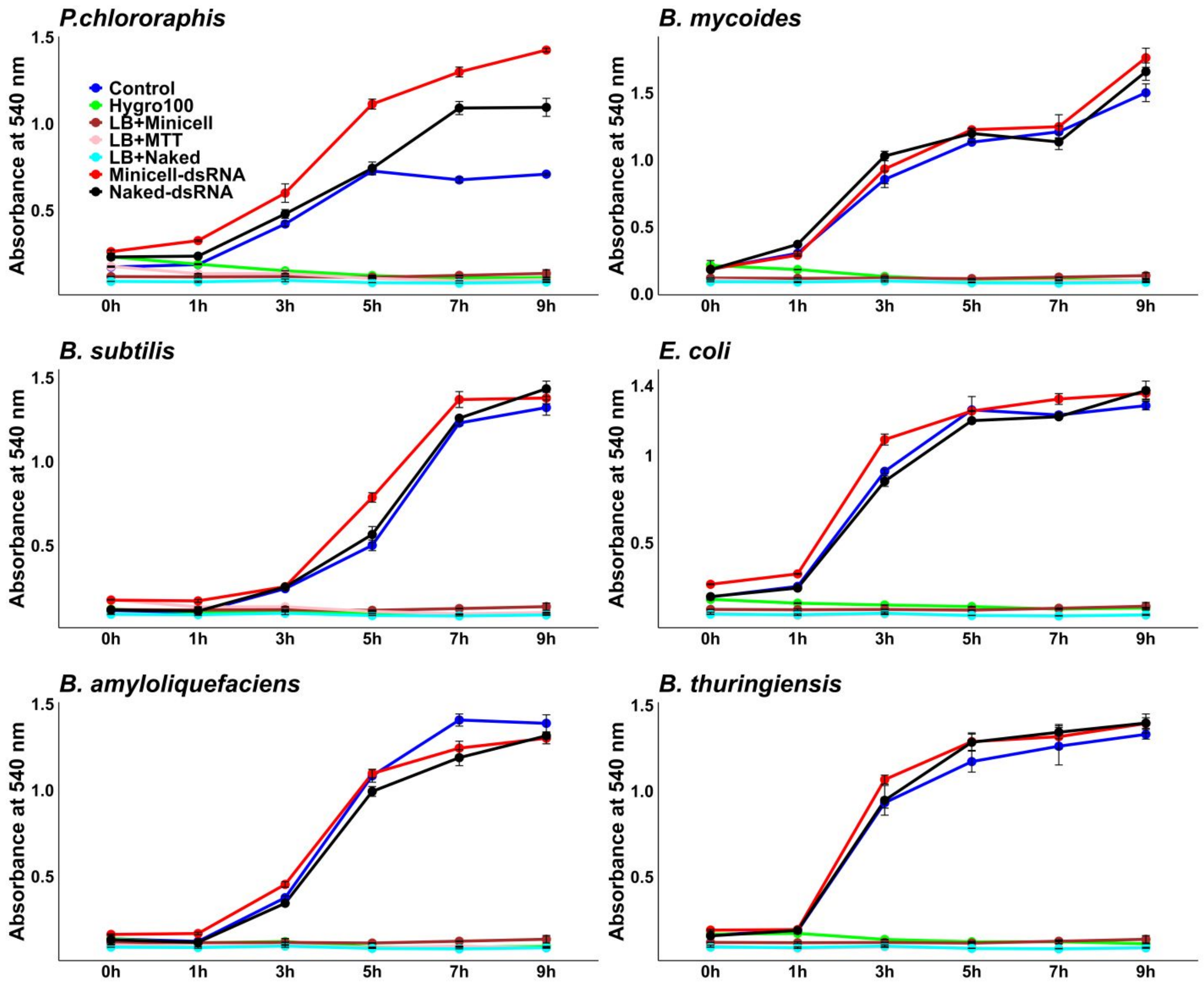


Figure S1: Growth kinetics of bacterial biocontrol agents exposed to various dsRNA formulations (Trial 1). Line plots illustrate the evolution of biocontrol agent populations over time, revealing the effects of Naked or minicell-based dsRNA treatments on growth dynamics.

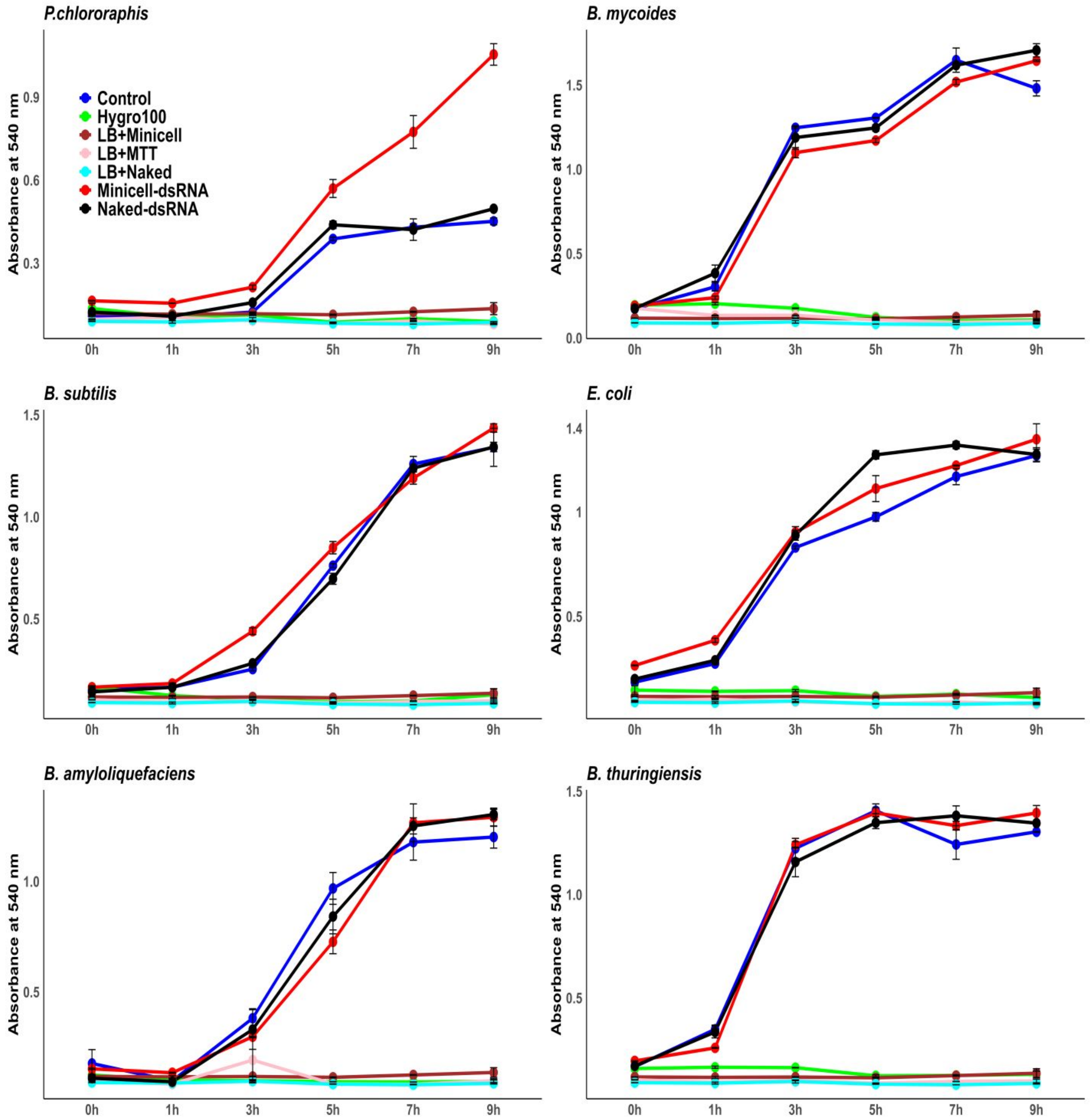


Figure S2: Growth kinetics of biocontrol agents exposed to various dsRNA formulations (Trial 2). Line plots illustrate the evolution of biocontrol agent populations over time, revealing the effects of Naked or minicell-based dsRNA treatments on growth dynamics.

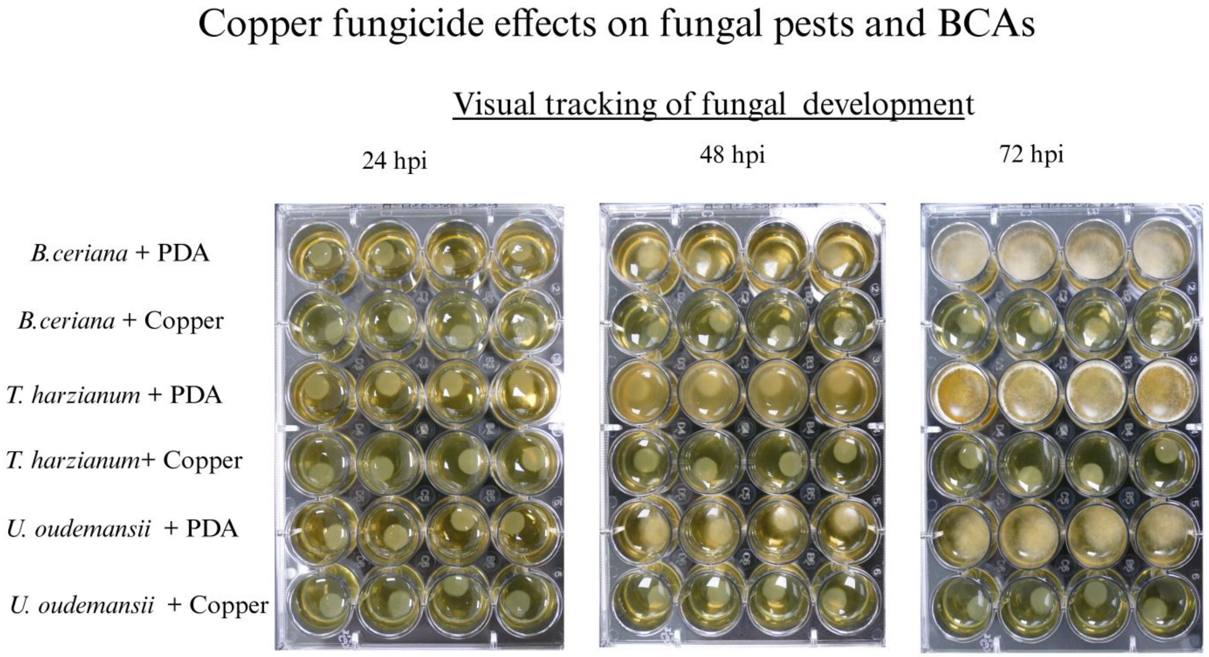


Figure S3: Assessment of copper fungicide (CUPROFIX® FLEX) specificity against two biocontrol agents (*T. harzianum* strain T-22 and *U. oudemansii* strain U3) and the fungal pathogen *B. cinerea*.

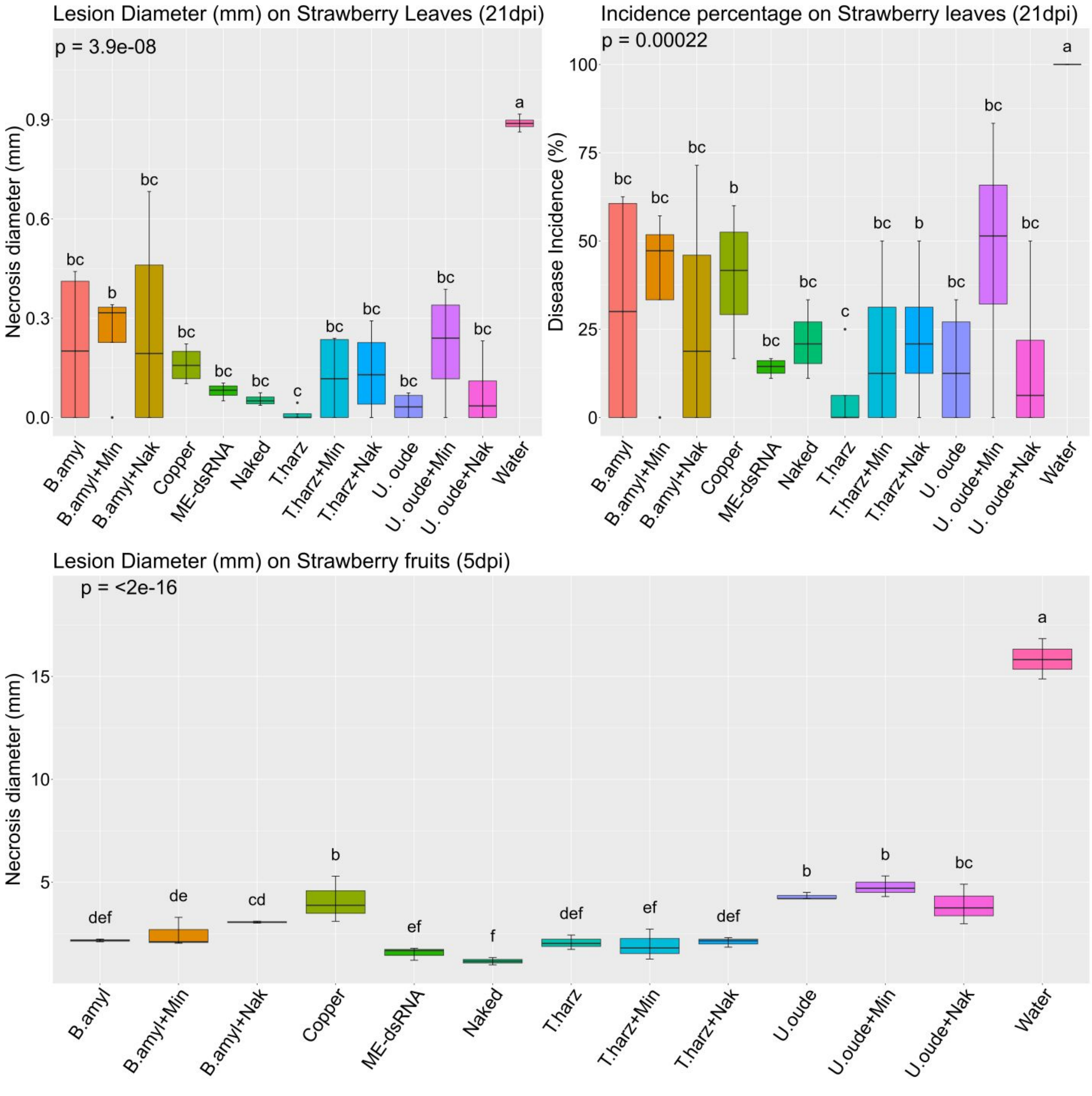


Figure S4: Comparative analysis of leaf necrosis and fruit infection diameter caused by *B. cinerea* on strawberries followed by naked (1000 ng/ml) and minicell-based dsRNA (2000ng/ml) treatment and BCAs, both individually and in combination (Trail 2). Box plots with identical letters indicate statistically insignificant.
